# Social Participation and Survival in Widowed Persons: Results of the Taiwan Longitudinal Study on Aging

**DOI:** 10.3390/ijerph182010974

**Published:** 2021-10-19

**Authors:** Yu-Han Hsiao, Meng-Chih Lee, Chih-Jung Yeh, Chi-Jung Tai, Shiuan-Shinn Lee

**Affiliations:** 1 Department of Public Health, Chung Shan Medical University, Taichung 40201, Taiwan; phoebe01026@gmail.com (Y.-H.H.); alexyeh@csmu.edu.tw (C.-J.Y.); 2 Department of Family Medicine, Taichung Hospital, Ministry of Health and Welfare, Taichung 40343, Taiwan; mengchihlee@gmail.com; 3 Institute of Medicine, Chung Shan Medical University, Taichung 40201, Taiwan; 4 Institute of Population Health Sciences, National Health Research Institutes, Miaoli 35053, Taiwan; 5 College of Management, Chaoyang University of Technology, Taichung 41331, Taiwan; 6 Department of Family Medicine, Pingtung Hospital, Ministry of Health and Welfare, Pingtung 90054, Taiwan; 7 Department of Family Medicine, Kaohsiung Medical University Hospital, Kaohsiung Medical University, Kaohsiung 80708, Taiwan

**Keywords:** social participation, survival, mortality, widowed persons

## Abstract

It has been considered that widowed persons have a higher risk of death. This study intended to explore whether social participation could improve this trend. A longitudinal study database was constructed to explore the trend of survival and its change with social participation in widowed persons. The Taiwan Longitudinal Study on Aging (TLSA), based on four consecutive waves of longitudinal follow-up data in 1999, 2003, 2007, and 2011 was linked with the National Death Registry from 1999 through 2012. In total, there were 1417 widowed persons and 4500 nonwidowed persons included in this study, excluding divorced and never-married people. The survival trend analysis was carried out with social participation as the main predictive factor stratified for comparative analysis. Our results showed that the widowed were older than the nonwidowed, were female-dominant, had a lower education level, were more economically stressed, and were less likely to engage in regular exercise, and thus showed generally poorer health; for example, being more vulnerable to having chronic diseases, disability with the Activities of Daily Living (ADL), cognitive impairment with the Short Portable Mental State Questionnaire (SPMSQ), and depression with The Center for Epidemiological Studies-Depression (CES-D). The death risk of the widowed was significantly higher than that of the nonwidowed, but the death trend for those with social participation was significantly lower than that of their counterparts in both the widowed and nonwidowed. After matching with gender and age for widowed persons, the widowed with social participation had a significantly lower risk of death (adjusted hazard ratio (HR), 0.83; 95% confidence interval (CI), 0.71–0.98) compared to the widowed without social participation. It was concluded that social participation can improve the death risk for the widowed, and it is worthily included in health promotion plans and social welfare services for widowed persons.

## 1. Introduction

Widowhood is a common phenomenon in an aging society [1,2]. According to the data released by Taiwan Department of Household Administration in 2021, by the end of 2020, the widowed aged 55–64 years old accounted for 5%, while the widowed over 65 accounted for 34%. Another characteristics of widowhood included being predominantly female and of older age, as the widowed rate of women over 65 years old accounted for 43.3%, which was much higher than that of 12% for men [3]. In general, the elderly are more likely to experience retirement, children leaving home, widowhood, and other adverse conditions in their later years. These events, particularly widowhood, may have a negative impact on their health and survival [4,5,6,7,8,9,10]. It has been suggested that the widowed have a higher risk of death [7,8,9,10,11], adverse health outcomes, and more stress in life than the nonwidowed [10,12,13,14]. The literature showed that in general, the widowed had excess mortality (compared with marriage), around 15% 3 months after the death of spouses [11]. In Taiwan, a previous study found that between 1999 and 2007, the widowed were 1.2 times more likely to die prematurely than married persons [8], and there were significant differences in gender and socioeconomic, mental, and physical status between widowed and nonwidowed persons [13,14,15]. The literature showed that the possible factors for the high risk of death in the widowed include physical and mental health, lifestyle, social support, and economic status [7,11,14,15]. 

It obviously is very important issue to determine how to improve the social determinants of care to improve the survival and health of the widowed. In particular, beyond unmodifiable factors such as age, gender, education level, race, chronic diseases, living environment, etc., we should focus on factors that can be changed, such as welfare services and social participation. Social participation is commonly defined as a person's involvement in activities that provide interaction with others in society or the community [16,17], and expresses interpersonal interactions outside the home, which is particularly important for the elderly and the widowed persons [18,19]. 

However, most previous studies did not analyze or follow up the longitudinal database, and only a few studies have addressed the direct effect of social participation on the health outcomes and survival among the widowed [16,19], most likely through increasing physical, psychological, and social vitality, and even obtaining more social support. The literature has shown that widowed persons are more predominantly female, older, and have more chronic diseases and mental disorders [13,14,15,20,21,22]. Even while knowing this, few remedial strategies have been suggested to improve the survival of widowed persons. Therefore, this study will extend the tracking time for more than 10 years using the Taiwan Longitudinal Study on Aging (TLSA), and conduct a comparative analysis of the widowed with and without social participation. We intended to determine the direct effect of social participation on the survival of the widowed, which can be used as a reference for health promotion plans and welfare policymaking and services. 

## 2. Methods

### 2.1. Data Source

The Taiwan Longitudinal Study on Aging (TLSA) is a population-based prospective cohort study that was initiated by the Health Promotion Administration, Ministry of Health and Welfare, Taiwan. This survey was first conducted in 1989 on older adult residents in nonaboriginal townships of Taiwan. A three-stage systematic random sampling design was used for the selection of an equal probability sample [23]. Data were collected through a face-to-face personal interview conducted by trained interviewers. Participants with significant cognitive impairments provided answers through their proxies. The respondents were later followed up every three to four years. Two fresh population samples were selected by the TLSA study group in 1996 and 2003 to maintain representativeness of the younger age cohort and to extend the representativeness of the sample to the population aged 50 and above. A total of seven surveys were conducted in 1989, 1993, 1996, 1999, 2003, 2007, and 2011 (Wave I–Wave VII, respectively). The details and design of the TLSA have been described elsewhere [23,24]. 

### 2.2. Study Group Identification, Study Design, and Ethical Approval

**Figure 1:**
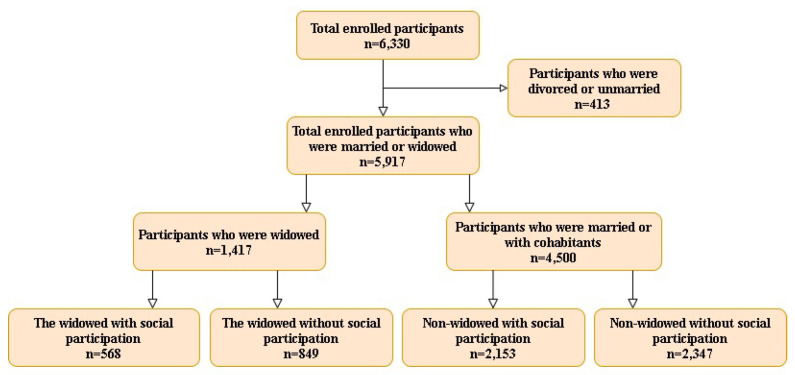
A flow diagram of the subject-inclusion process.

In this study, we analyzed 6330 sample subjects aged ≥ 50 years old in the 1999 through 2011 study waves. Individuals who were unmarried or divorced, totaling 413, were excluded from the study (Figure 1). Thus, a total of 5917 subjects were included in the final analysis, including 1417 widowed persons and 4500 nonwidowed persons. The current study was approved by the Institutional Review Board of Health Promotion Administration, Ministry of Health and Welfare (Approval No. BHP-2007–002).

### 2.3. Research Variables

#### 2.3.1. Independent Variable: Social Participation

Social participation as the main predictor was defined as whether the individual was a member of or had currently joined activities of a variety of groups, including professional, social, relative, religious, recreational, political, elderly, or local community residential groups [25]. The individual was defined and allocated to the social participation group if they answered yes to whether they participated in any of the above-mentioned groups. If not, the individual was allocated to the non-social-participation group [25].

#### 2.3.2. Dependent Variable: Mortality

This study examined the effect of social participation on the mortality of older adults in Taiwan. Therefore, mortality was the only dependent variable. It was measured in survival years estimated starting from 1999 to 2012 using the Taiwan National Death Registry (TNDR) record, which provides survival status and date of death. The participants’ national identification number was used to link the data from TLSA and TNDR databases in each of the follow-up surveys.

#### 2.3.3. Covariates

The gathered subject data comprised age, gender, level of education (uneducated, elementary school, junior high school, senior high school, and college or above), marital status (widowed and nonwidowed persons), self-reported health, self-perceived economic pressure, smoking, alcohol consumption, and history of major diseases including hypertension, diabetes mellitus (DM), cardiovascular disease, chronic obstructive pulmonary disease (COPD), history of stroke, and history of cancer.

Functional status included the ability to perform the Activities of Daily Living (ADL) including bathing, dressing, eating, transferring from bed, walking, and toileting [26], and we again regrouped the subjects into two groups: none and at least one ADL disability [27]. Cognitive function was measured by the nine-item Short Portable Mental Status Questionnaire (SPMSQ) [28] in 1999. With total scores ranging from 0 to 9, correct answers were coded 0, whereas errors were coded 1 [29]. In this study, the questions regarded where the subject was currently located; their home address; the current day, month, and year; their age; and the names of the current and last presidents. Participants with four or more errors were defined as having cognitive impairment, which has been frequently adopted in several cohort studies that support this cut-off point [30]. The 10-item CES-D identified depressive symptoms, the scores for which ranged from 0 to 30, with a cut-off point for the definition of depression of 8 or more [31].

### 2.4. Measures

#### Statistical Methods

A Kaplan–Meier curve based on Taiwan’s National Death Registry database linked with our study subjects was made for subjects that were widowed or nonwidowed, and also was further stratified by whether or not there was social participation. Survival differences were evaluated using the logrank test. Bivariate and multivariate analyses were conducted using a multivariate Cox proportional hazards regression model, and the hazard ratio (HR) and 95% confidence interval (CI) were calculated [32]. In the stratified analysis, we divided the widowed into two groups: with or without social participation. Considering that gender and age were important confounding factors for the direct effect of social participation on the death risk trend of the widowed [4,7,8,9,11,24], after we divided the widowed into two groups (with or without social participation), they underwent 1:1 propensity-score (PS) matching by age and gender, which might also have covered the effect of related physical health confounders such as ADL and SPMSQ. After PS matching with gender and age, the two most potential confounders for mortality in widowed persons, we also adjusted the hazard ratio for certain background characteristics, educational level, and various health status measures in the multivariate Cox regression analysis. Since the educational level had been included, its highly related variable of self-perceived economic pressure was not included in the model.All statistical tests were carried out as two-sided, and significant differences were considered at *p* < 0.05. Statistical analyses were conducted using SAS version 9.4 (SAS Institute Inc., Cary, NC, USA).

## 3. Results

### 3.1. Demography

There were a total of 5917 subjects included in this study, and 24.0% of them were widowed. The characteristics and examination of the differences between the widowed and nonwidowed are shown in Table 1. Statistically significant differences between the two groups were found in social participation, age, education level, self-reported health, self-perceived economic pressure, health behaviors, comorbidities, physical dysfunction, and depressive symptoms. Widowed persons were more likely to be older, have a lower education level, have poor self-reported health, perceive more financial pressure, have higher rates of chronic diseases, have higher rates of stroke and cancer, and were more likely to be judged as disabled by ADL, cognitive impairment by SPMSQ, and own depressive symptoms by CES-D.

### 3.2. Mortality

**Figure 2:**
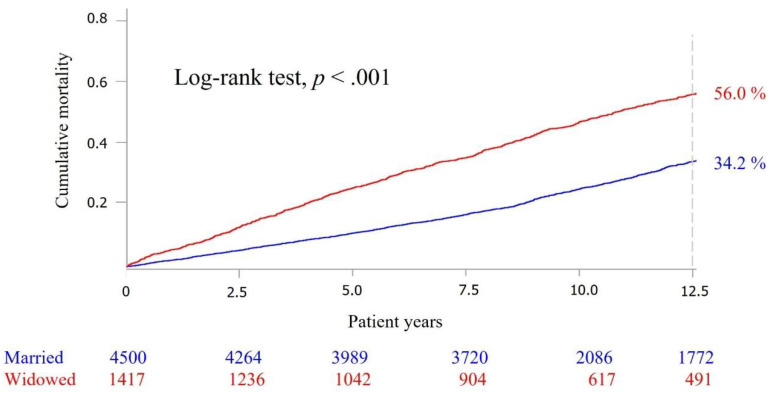
Kaplan–Meier survival curve between the widowed (red line) and nonwidowed (blue line).

**Figure 3:**
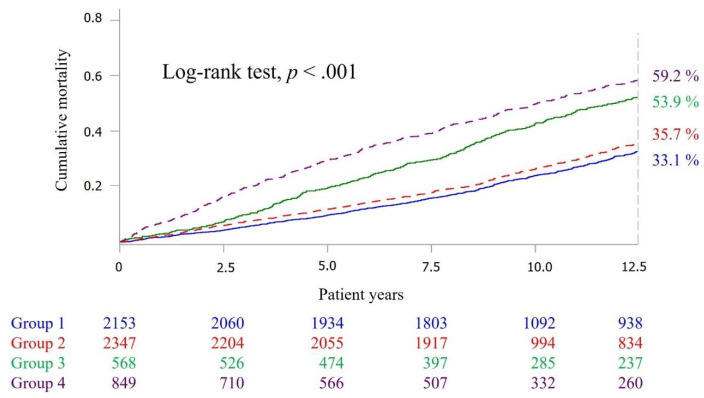
Kaplan–Meier survival curves among groups. Group 1: nonwidowed with social participation; Group 2: non-widowed without social participation; Group 3: widowed with social participation; Group 4: widowed without social participation.

Kaplan–Meier (KM) survival curves were used to present the survival curves of the widowed and the nonwidowed, and showed that the death risk of the widowed was higher, and was significantly different (*p* < 0.001) from that of the nonwidowed (Figure 2). When the death risk trend presented by the survival curve for the widowed and nonwidowed was stratified by social participation, the widowed without social participation had the highest death risk, followed by the widowed with social participation, nonwidowed with social participation, and nonwidowed with social participation. There was a significant difference (*p* < 0.001) in the death risk trend among the four survival curves as examined by the logrank test (Figure 3).

Taking the mortality of the nonwidowed with social participation as the reference group, the widowed without social participation had the highest risk of death (HR, 2.35; 95% CI, 2.09–2.64), followed by the widowed with social participation, and the nonwidowed without social participation (Table 2). This showed that the death risks of both the widowed with social participation group and the widowed without social participation group were significantly higher than that of the reference group, and the test for trend showed a significant difference (*p* < 0.001).

### 3.3. Matching Analysis in the Widowed

In order to explore the direct effect of social participation on the death risk for the widowed, this study first checked the indirect effect of age and gender on the death risk. An odds ratio (OR) of 3.55 (95% CI 2.52–4.99, *p* < 0.001) was found when comparing the widowed over 65 years old to those under 65 years old by using multivariate logistic regression. As for the gender, the odds ratio of death for men to women was 2.86 (95% CI 2.05–3.98, *p* < 0.001). Considering that gender and age are important confounding factors for the direct effect of social participation on the death risk trend of the widowed, this study divided the widowed into two groups (with or without social participation), and underwent 1:1 matching for both age and gender. The baseline characteristics of matched widowed participants with and without social participation are shown in Table 3, and no difference was found in age, gender, and a variety of physical and mental conditions between the two groups after matching.

With adjustment of educational level and comorbidities in the multivariate Cox regression model, and the adjusted hazard ratio for social participation to non-social-participation after matching for age and gender, the death risk of the widowed with social participants was significantly lower than for the widowed without social participation (adjusted hazard ratio 0.83; 95% CI 0.71–0.98) (Table 4).

## 4. Discussion

Widowed persons had a higher risk of death for many possible reasons; for example, age, gender, economic pressure, and physical and mental conditions [13,14,15,20,21,22,24]. Unfortunately, most of these are unmodifiable. For example, in terms of age and gender, this study found that the widowed were older and female-dominant, and these two factors still brought a higher risk of death even under the control of other covariables [12,24,33,34]. Faced with these two major confounders for the death risk of the widowed, this study was designed to examine the direct effect of social participation on the death risk of the widowed. After matching by age and gender, it was also clearly found that social participation could indeed reduce the risk of death of the widowed.

Our study found that the widowed with social participation had a lower risk of death compared to their counterparts, which was consistent with previous studies [35,36,37,38,39,40,41,42]. Previous studies have shown that social participation can increase physical, psychological, and social vitality, and even obtains more social support, whether emotional or instrumental social support [35,36]. Combining these favorable effects can improve the loneliness and depressive symptoms [37], delay cognitive impairment [38,39], and promote self-perceived health [40,41] and daily activity functions [42] of the widowed. Therefore, efforts should be made to promote social participation for the widowed, which can reduce the above-mentioned undesirable results caused by widowhood, and lead to a decrease in the death risk. The current common practice may begin with participating in local community residential activities for the elderly. In Taiwan, there are many activities for the elderly at various community activity centers or community spots (a total of 3149 settings in Taiwan, nearly 1 for each neighborhood in city or village in rural areas) for the elderly to undergo cultural, physical, social, and recreational activities sponsored by the Ministry of Health and Welfare. The current participation rate is about 8.7% for the elderly aged 65 or above in 2020 [43]. However, these efforts have still not focused on the widowed, physically and mentally handicapped, and the elderly who are living alone.

The mechanism of why social participation reduces the risk of death of the widowed remains to be discussed. At present, it is considered that it may increase physical activity [42], reduced depressive symptoms [37], and increased social capital or social support [35,36]. Previous studies have found that social participation reduces adverse outcomes by increasing social support, including emotional and instrumental social support [16,19,25,35,36]. We hypothesize and recommend that social participation can reduce the risk of death by increasing emotional and instrumental social support. Furthermore, the direct and indirect effects on the quantities such as the frequency and the intensity, and on the qualities such as category and role of social participation, on health outcomes and death risk could be the direction of future research. Indeed, having a job, whether a full- or part-time job, has been shown to be beneficial in reducing mortality for the elderly [44], and could be also included in health promotion plans for widowed persons.

There were some limitations to this study. For example, all data in this study were self-reported through face-to-face interviews, so a reporting bias might be present, although the instruments utilized in this study were verified in previous studies [24,35,45]. In addition, biomedical data from laboratory tests to predict mortality were not available. Moreover, no standardized assessment including quantitative and qualitative variables of social participation has yet been established for evaluation of its effects on mortality. On the other hand, the TLSA has always obtained a high response rate, with a small amount of missing data, and enough of a representative sample in all waves, which may have compensated for the above shortcomings. The TLSA is a longitudinal follow-up study that was also helpful in eliminating the possibility of reverse causality. In this study, we used subjective assessment for variables such as self-reported health or self-perceived economic pressure level, which may have been influenced by mood states such as depression or poor cognitive function in older persons. In addition, those who had a lower educational level consistently reported poorer conditions compared with their counterparts in previous studies [46]. Future research should take more social determinants such as socioeconomic status (SES), the social security system, health care services, leisure activities, and employment, as well as physical and mental indicators, into consideration.

## 5. Conclusions

We concluded that social participation could improve the death risk for widowed persons through increasing physical, psychological, and social vitality, and even obtaining more social support during social participation. Therefore, it is worth encouraging social participation in health promotion programs and social welfare services in the elderly, especially for the widowed.

## Figures and Tables

**Table 1:** Baseline demographics of participants who were widowed, married, and with cohabitants from the Taiwan Longitudinal Study on Aging (TLSA) waves IV–VI (1999–2011).

	Widowed *n* = 1417		Nonwidowed *n* = 4500		*p* Value
	N	%	N	%	
**Age, years**	69.5 ± 10.7		60.3 ± 8.0		<0.001 ^†^
Male	382	27.0	2640	58.7	<0.001 *
**Social participation**					<0.001 *
No	849	60.0%	2347	52.2%	
Yes	568	40.0%	2153	47.8%	
**Age group**					<0.001 *
50 ≤ age < 65	933	65.8	3642	80.9	
65 ≤ age < 70	116	8.2	455	10.1	
70 ≤ age < 75	90	6.4	180	4.0	
Age ≥ 75	278	19.6	223	5.0	
**Education level**					<0.001 *
Illiterate	635	44.8	847	18.8	
Elementary school	596	42.0	2130	47.3	
Junior high school	90	6.4	555	12.3	
Senior high school	66	4.7	521	11.6	
College or above	30	2.1	447	9.9	
**Urbanization of residence**					<0.001 *
Nonrural areas and offshore islands	1355	95.6	4375	97.2	
Rural areas and offshore islands	62	4.4	125	2.8	
**Self-rated health**					<0.001 *
Good or very good	377	26.6	1997	44.4	
Okay	467	33.0	1418	31.5	
Bad or very bad	573	40.4	1085	24.1	
**Self-reported economic pressure**					<0.001 *
No	822	58.0	2736	60.8	
Mild	437	30.8	1582	35.1	
Severe	158	11.2	182	4.1	
**Healthy and unhealthy behaviors**					
Exercise	724	51.1	2499	55.5	<0.001 *
Smoking	203	14.3	1195	26.6	<0.001 *
Alcohol	216	15.2	1416	31.5	<0.001 *
**Comorbidities**					
Hypertension	519	36.6	1283	28.5	<0.001 *
Diabetes mellitus	214	15.1	550	12.2	<0.001 *
Heart diseases	318	22.4	584	13.0	<0.001 *
Stroke	112	7.9	193	4.3	<0.001 *
Cancer	46	3.3	122	2.7	<0.001 *
Bronchitis	186	13.1	465	10.3	<0.001 *
**Physical and psychological function tests ^a^**					
ADL disability	223	15.7	227	5.0	<0.001 *
Cognitive impairment by SPMSQ	201	14.2	304	6.8	<0.001 *
Depression by CES-D	478	33.7	731	16.2	<0.001 *

ADL, activities of daily living; CESD, Center for Epidemiologic Studies Depression; SPMSQ, short portable mental status questionnaire. ^a^ The groupings were identified by ADL scale, SPMSQ, and CESD with validated cutoff values. Details are available in Materials and Methods section of the article. * Chi-square test, *p* < 0.05. † Student's t-test, *p* < 0.05.

**Table 2:** Mortality and hazard ratios among groups.

	Mortality	HR	95% CI	*p* Value	*p* for Trend
Group 1	651 (30.2%)	1.00	-	-	*p* < 0.001 *
Group 2	740 (31.5%)	1.11	0.99–1.23	0.05	
Group 3	307 (54.1%)	1.92	1.67–2.20	<0.001 *	
Group 4	491 (57.8%)	2.35	2.09–2.64	<0.001 *	

CI, confidence interval; HR, hazard ratio. Group 1: nonwidowed with social participation; Group 2: nonwidowed without social participation; Group 3: widowed with social participation; Group 4: widowed without social participation. * A *p*-value < 0.05 was considered significant.

**Table 3:** Baseline characteristics of matched widowed participants with and without social participation.

	Widowed with Social Participation *n* = 529		Widowed without Social Participation *n* = 529		*p* Value
	N	%	N	%	
**Age, years**	68.4 ± 10.0		68.0 ± 11.2		0.649
Male	157	29.7%	157	29.7%	1.000
**Age group**					1.000
50 ≤ age <65	368	69.6%	368	69.6%	
65 ≤ age <70	50	9.5%	50	9.5%	
70 ≤ age <75	34	6.4%	34	6.4%	
Age ≥ 75	77	14.6%	77	14.6%	
**Education level**					0.648
Illiterate	222	42.0%	227	42.9%	
Elementary school	221	41.8%	232	43.9%	
Junior high school	38	7.2%	35	6.6%	
Senior high school	33	6.2%	24	4.5%	
College or above	15	2.8%	11	2.1%	
**Urbanization of residence**					0.466
Nonrural areas and offshore islands	502	94.9%	507	95.8%	
Rural areas and offshore islands	27	5.1%	22	4.2%	
**Self-rated health**					0.043 *
Good or very good	156	29.5%	130	24.6%	
Okay	189	35.7%	177	33.5%	
Bad or very bad	184	34.8%	222	42.0%	
**Self-reported economic pressure**					<0.001 *
No	357	67.6%	281	53.1%	
Mild	148	28.0%	169	32.0%	
Severe	24	4.5%	79	14.9%	
**Healthy and unhealthy behaviors**					
Exercise	318	60.1%	244	46.2%	<0.001 *
Smoking	80	15.1%	87	16.5%	0.555
Alcohol	83	15.7%	86	16.3%	0.791
**Comorbidities**					
Hypertension	185	35.0%	195	36.5%	0.506
Diabetes mellitus	68	12.9%	75	14.2%	0.529
Heart diseases	131	24.8%	106	20.0%	0.070
Stroke	27	5.1%	47	8.9%	0.016 *
Cancer	11	2.1%	21	4.0%	0.073
Bronchitis	81	15.3%	64	12.1%	0.132
**Physical and psychological function tests ^a^**					
ADL disability	45	8.5%	97	18.3%	<0.001 *
Cognitive impairment by SPMSQ	64	12.1%	67	12.7%	0.298
Depression by CES-D	153	30.5%	185	41.6%	0.035 *

ADL, activities of daily living; CESD, Center for Epidemiologic Studies Depression; SPMSQ, short portable mental status questionnaire. ^a^ The groupings were identified by ADL scale, SPMSQ, and CESD with validated cutoff values. Details are available in Materials and Methods section of the article. * Chi-square test, *p* < 0.05.

**Table 4:** Social participation and mortality in the widowed after matching with age and gender.

	Mortality N (%)	Adjusted HR ^#^	95% CI	*p*-Value
Widowed without social participation (*n* = 529)	295 (55.6%)	1.00	-	-
Widowed with social participation (*n* = 529)	286 (54.1%)	0.83	0.71–0.98	0.04 *

^#^ Adjusted for educational level and comorbidities including hypertension, diabetes, and heart diseases. * A *p*-value < 0.05 was considered significant.

## Data Availability

The datasets generated during the current study are not publicly available, but data are however available from the applicants upon reasonable request and with permission of the Ministry of Health and Welfare in Taiwan.
